# Assessment of left ventricular geometrical patterns and function among hypertensive patients at a tertiary hospital, Northern Tanzania

**DOI:** 10.1186/1471-2261-12-109

**Published:** 2012-11-23

**Authors:** Lairumbe Korduni Silangei, Venance Philis Maro, Helmut Diefenthal, Gibson Kapanda, Matthew Dewhurst, Hery Mwandolela, Ben Hamel

**Affiliations:** 1 Kilimanjaro Christian Medical University College, P. O. Box 2240, Moshi, Tanzania; 2 Kilimanjaro Christian Medical Centre, P. O. Box 3010, Moshi, Tanzania; 3 Heameda Medical Clinic, P. O. Box 12633, Dar es Salaam, Tanzania; 4 Freeman Road Hospital, Newcastle upon Tyne, NE7 7DN, UK; 5 Dar es Salaam University College of Education, P. O. Box 2329, Dar es Salaam, Tanzania

**Keywords:** Echocardiography, Essential hypertension, Left ventricular hypertrophy, Left ventricular geometry, Left ventricular mass index, Left ventricular function

## Abstract

**Background:**

With hypertension, the cardiovascular system changes to adapt to the varying neuro-humoral and hemodynamic changes and this may lead to the development of different left ventricular geometric patterns, each carrying a different risk profile for major adverse cardiovascular events.

**Methods:**

Using a consecutive sampling technique, a cross-sectional, prospective, hospital based study was done and two hundred and twenty seven (227) hypertensive patients were studied.

**Results:**

The distribution of different abnormal LV geometrical patterns was 19.8%, 28.2%, 22% for concentric remodelling, concentric hypertrophy and eccentric hypertrophy respectively. With echocardiographic criteria, the proportion of patients with left ventricular hypertrophy (LVH) was higher when left ventricular mass (LVM) was indexed to height^2.7^ than to body surface area (70.0% vs. 52.9%). Duration of hypertension markedly influenced the type of LV geometry with normal LV geometry predominating in early hypertension and abnormal geometrical patterns predominating in late hypertension. The left ventricular fractional shortening decreased with duration of hypertension and was common in patients with eccentric hypertrophy. Age of the patient, systolic blood pressure, duration of hypertension and body mass index were found to be independent predictors left ventricular hypertrophy.

**Conclusion:**

About 70% of hypertensive patients had abnormal geometry existing in different patterns. Eccentric hypertrophy had more of clinical and echocardiographic features suggestive of reduced left ventricular systolic function. Hypertensive patients should be recognized as a heterogeneous population and therefore stratifying them into their respective LV geometrical patterns is useful as way of assessing their risk profile as well as instituting appropriate management.

## Background

The World Health Organization (WHO) reported that non-communicable diseases accounted for 60% of the world's total deaths, and about 80% of these are occurring in the developing countries [1]. In Africa, cardiovascular disease is no longer an emerging problem, but has become firmly established and its magnitude is approaching that of an epidemic [2].

There are many conditions that are known to increase risk of cardiovascular diseases but hypertension has long been known explicitly to be a strong and independent risk factor for the development of major adverse cardiovascular events, whereby the cardiovascular system adopts different abnormal changes as a result of long standing hypertension [3]. The heart adaptation to these changes includes geometrical re-orientation in different patterns notably concentric remodelling, concentric hypertrophy or eccentric hypertrophy [4]. The type of geometric pattern is determined mainly by the predominating type of stressor/stimulus to the myocardium, i.e. volume overload or pressure overload [5,6]. With time due to unknown factors these changes become maladaptive and predispose patients to major adverse cardiovascular events. The risks vary from one patient to another depending on the type and severity of change.

World Health Organization (WHO) defines hypertension as a chronic medical condition in which the arterial blood pressure is elevated ≥140 mmHg systolic and/or ≥90 mmHg diastolic on two readings taken apart or a reported diagnosis of hypertension and treatment with recognized anti- hypertensive within 2 weeks before the visit. In a cross-sectional study involving three Sub-Saharan African countries (Tanzania, Malawi and Rwanda), together, the prevalence of hypertension and pre-hypertension was found to be 22 and 44% respectively [7].

The definition and criteria of left ventricular hypertrophy (LVH) are variable in many studies [8]. The diagnosis of LVH has been incorporated in clinical practice as an important marker of cardiovascular disease. Its prevalence depends on classification criteria and specific population characteristics, ranging from 3% in normotensive community based samples [9] to about three-quarters of hypertensive patients [10].

## Methods

### Study population

The study population included adult patients firmly diagnosed to have essential arterial hypertension with or without treatment. A consecutive sampling method was used. We excluded patients who had poor quality of echocardiography imaging due to various reasons including obesity and lung problems. Patients noted to have impaired global or segmental wall motion and valvular lesions during echocardiography examination were excluded. Also patients with suspected or established secondary hypertension were also excluded.

### Study procedures

During recruitment, the principal investigator (PI) filled in a standardized structured questionnaire for each patient included in the study. A detailed history was obtained from the patient or from a close relative and recorded in the patient case-note and in the structured questionnaire. Physical examination findings, electrocardiogram and echocardiogram results were recorded in the data collection sheet.

### Patient evaluation: history and physical examination

A thorough and detailed history was taken and in particular the history focused on presence or absence of overt heart-related symptoms. Duration of hypertension was also noted.

Socio-demographic data, anthropometric and biometric measurements were taken: – Height (in meters) was taken using a commercially available stadiometer and the patient was required to stand upright away from but body and head touching the meter ruler (m). Weight (in kilograms) was taken using a standard weighing scale to the nearest rounded value while the patient is in light clothing and shoes taken off.

A thorough general examination was conducted to each patient and vital signs obtained. Blood pressure measurement using standard well calibrated mercury sphygnomanometer based was taken.

The body mass index (BMI) was derived by dividing the weight (kg) by the square of the height (m) and classification of obesity was done according to WHO criteria. The body surface area (BSA) was calculated using the formula derived by Du Bois and Du Bois (1929) as: BSA = (Weight^0.425^ × Height^0.725^) × 0.007184.

The rest of body systems were examined and findings recorded in the patient case note and the questionnaire.

### Echocardiography

2D- and M-Mode echocardiography was performed using a commercially available Philips/Agilent 5500 Sonos machine with a 3.25-MHz transducer, in subjects in supine left lateral position and an average of 3 values/readings were taken as per the American Society of Echocardiography (ASE) recommendations. The M- mode derived parasternal view was used to assess for the chamber and wall dimensions of the left ventricle and left atrium, including the derived systolic indices such as fractional shortening.

The ASE recommended formula-leading edge to leading edge measurements-for estimation of Left Ventricular mass (LV mass) from left ventricular linear dimensions [5] was used, whereby:

(1) $$LVmass=0.8\times (1.04\left[{\left(LVIDd+LVPWd+IVSd\right)}^{3}-{\left(LVIDd\right)}^{3}\right]+0.6g$$

Whereby,

1. LVPWd – posterior wall thickness at end diastole.

2. IVSd – septal wall thickness at end diastole.

3. LVIDd – dimensions of the left ventricle at end-diastole

Gender-specific and indexation of left ventricular mas (LVM) was used to diagnose LVH using the following defining criteria: >115g/m^2^ and >95g/m^2^ for men and women respectively when LVM was indexed to body surface area (BSA) and >49.2g/m^2.7^ and 46.9 g/m^2.7^ for men and women respectively, when LVM was indexed to allometric growth rate of 2.7. The relative wall thickness (RWT) was calculated using the formula 2 × LVPWd/LVIDd. Increased RWT was present when this ratio was >0.42 [5].

Left ventricular (LV) geometry was divided into four patterns based on left ventricular mass index (LVMI) and relative wall thickness (RWT) values. These are:

1. Normal geometry (NG) – normal LVMI and RWT ≤ 0.42,

2. Concentric hypertrophy (CLVH) – increased LVMI and RWT > 0.42,

3. Eccentric hypertrophy (ELVH) – increased LVMI and RWT ≤ 0.42,

4. Concentric remodelling (CR) – normal LVMI and RWT > 0.42.

### Statistical analysis

SPSS version 17.0 software (SPSS, Chicago, IL, USA) was used in the analysis of the data. Continuous variables were expressed as mean ± SD while categorical variables were expressed as counts (percentages). Chi-square (χ2) analysis was done to compare proportions. Logistic regression was run to determine predictors of LVH. A 2-tailed p-value of 0.05 was taken as statistically significant.

### Ethical clearance and consent

This study was ethically approved by the Kilimanjaro Christian Medical University College (KCMUCo) Research Ethics Committee after meeting the required ethical standards.

The principal investigator fully explained to every prospective participant the purpose of the research and was requested to participate freely. A consent form containing details of the study regarding main purpose, role of participants, and benefits associated with participating in the study (if any) was given to each prospective participant. Those who agreed to participate in the study gave a verbal consent as well as signing a written consent form written both in English and Swahili language. Swahili language is a lingua franca in Eastern Africa.

Patients who did not agree to participate in the study were not denied any services or treated with partiality and even those who agreed to be recruited in the study were not treated differently from those who did not agree to participate in the study.

## Results

### Clinical characteristics of hypertensive according to duration of hypertension

As shown in Table 1, two hundred and twenty seven hypertensive patients (84 males and 143 females) participated in this study. The mean age was 61years with standard deviation of 11.3. The proportion of patients according to duration of hypertension significantly increased with age (p <0.001). Duration of hypertension was arbitrarily defined as early it was present for less than 1 year, intermediate if it is diagnosed between 1 but less than 5 years and long term if the hypertension was diagnosed and present for 5 years or more. Mean age of early hypertension was 55.3 years, increasing to 59.6 years for intermediate to 65.8 years for long-term hypertension. Duration of hypertension was independent of sex, body surface area, body mass index, systolic blood pressure, diastolic blood pressure and mean arterial pressure.

**Table 1:** Clinical characteristics of hypertensive patients according to duration of hypertension

**Clinical parameter**	**All (n=227)**	**Duration of hypertension**			**p value**
		**Early (n=44)**	**Intermediate (n=101)**	**Long term (n=82)**	
Age (years ± SD)	61.0 ± 11.3	55.3 ± 10.9	59.6 ± 10.5	65.8 ± 10.8	<0.001
Age group: < 50 years [n(%)]	38 (16.7)	14 (36.8)	18 (47.4)	6 (15.8)	<0.001
Age group: 50 – 69 years [n(%)]	127 (55.9)	24 (18.9)	63 (49.6)	40 (31.5)	
Age group: ≥70 years [n(%)]	62 (27.3)	6 (9.7)	20 (32.3)	36 (58.1)	
Male sex [n(%)]	84 (37.0)	18 (21.4)	32 (38.1)	34 (40.5)	0.331
Female sex [n(%)]	143 (63.0)	26 (18.2)	69 (48.3)	48 (33.6)	
BMI (kg/m^2^ ± SD)	27.6 ± 5.3	27.5 ± 5.1	27.5 ± 5.6	27.8 ± 5.1	0.91
BSA (m^2^ ± SD)	1.75 ± 0.17	1.79 ± 0.20	1.74 ± 0.16	1.73 ± 0.16	0.129
SBP (mmHg ± SD)	161.2 ± 19.08	157.7 ± 17.50	162.57 ± 8.09	161.22 ± 20.99	0.373
DBP (mmHg ± SD)	97.27 ± 10.41	98.64 ± 9.77	97.43 ± 11.46	96.34 ± 9.36	0.491
PP (mmHg ± SD)	63.88 ± 16.59	59.09 ± 4.28	65.15 ± 16.53	64.88 ± 17.52	0.102
MAP (mmHg ± SD)	118.56 ± 1.51	118.33 ± 0.99	119.14 ± 11.66	11.97 ± 11.71	0.783

BMI=Body Mass Index, BSA=Body Surface Area, SBP=Systolic Blood Pressure, DBP=Diastolic Blood Pressure, PP=Pulse Pressure, MAP=Mean Arterial Blood Pressure.

### Echocardiographic characteristics of hypertensive patients according to duration of hypertension

As shown in Table 2, all mean values of echocardiographic parameters were significantly influenced by duration of hypertension as they increased in dimensions with duration of hypertension (p<0.001). However, left ventricular fractional shortening decreased with duration of hypertension (p=0.003).

**Table 2:** Echocardiographic characteristics of hypertensive patients according to duration of hypertension

**Parameter value ±SD**	**All (n=227)**	**Duration of hypertension**			**p value**
		**Early**	**Intermediate**	**Long term**	
		**(n=44)**	**(n=101)**	**(n=82)**	
IVSWTd (cm)	1.20 ± 0.29	1.03 ± 0.26	1.18 ± 0.29	1.32 ± 0.26	<0.001
LVPWTd (cm)	1.09 ± 0.27	0.95 ± 0.23	1.09 ± 0.25	1.18 ± 0.27	<0.001
LVIDd (cm)	4.86 ± 0.46	4.73 ± 0.33	4.75 ± 0.41	5.07 ± 0.49	<0.001
LVIDs (cm)	3.38 ± 0.50	3.21 ± 0.38	3.27 ± 0.43	3.61 ± 0.56	<0.001
FS (%)	30.88 ± 5.53	32.41 ± 5.21	31.50 ± 5.22	29.29 ± 5.76	0.003
RWT(cm)	0.45 ± 0.11	0.40 ± 0.09	0.46 ± 0.09	0.47 ± 0.14	0.004
LVM(g)	218.40 ± 88.24	169.18 ± 72.95	208.59 ± 90.93	256.95 ± 75.85	<0.001
LVMI-BSA (g/m^2^)	125.90 ± 52.95	94.59 ± 40.38	120.39 ± 55.26	149.49 ± 45.32	<0.001
LVMI-height^2.7^ (g/m^2.7^)	61.56 ± 27.66	45.50 ± 21.81	58.80 ± 29.22	73.59 ± 23.08	<0.001

IVSWTd = Inter-ventricular septal wall thickness at end-diastole, LVPWTd=Left ventricular posterior wall thickness at end-diastole, LVIDd=Left ventricular internal diameter at end-diastole, LVIDs=Left ventricular internal diameter at end-systole, FS= Fractional Shortening, RWT=Relative wall thickness, LVM=Left ventricular mass, LVMI-BSA= Left ventricular mass indexed to body surface area, LVMI-height^2.7^= Left ventricular mass indexed to height^2.7^.

LV normal geometry was detected in 68 (30.0%) of the 227 hypertensive patients, concentric hypertrophy in 64 (28.2%), concentric remodelling in 45 (19.8%) and eccentric hypertrophy in 50 (22.0%).

### LV geometrical patterns according to duration of hypertension

In Table 3, it is shown that normal LV geometry was present in majority of patients with early hypertension 28 (63.6%) while eccentric hypertrophy was least in patients with intermediate hypertension (11.9%) and highest in patients with long term hypertension (43.9%). The difference between LV geometrical patterns and duration of hypertension was significant (p<0.001).

**Table 3:** Left Ventricular geometrical patterns according to duration of hypertension

**LV geometrical patterns**	**All (n=227)**	**Duration of hypertension**			**p value**
		**Early (n=44)**	**Intermediate (n=101)**	**Long term (n=82)**	
NG [n(%)]	68 (30.0)	28 (63.6)	32 (31.7)	8 (9.8)	<0.001
CR [n(%)]	45 (19.8)	10 (22.7)	27 (26.7)	8 (9.8)	
CH [n(%)]	64 (28.2)	4 (9.1)	30 (29.7)	30 (36.6)	
EH [n(%)]	50 (22.0)	2 (4.5)	12 (11.9)	36 (43.9)	

NG=normal geometry, CR=concentric remodelling, CH=concentric hypertrophy, EC=Eccentric hypertrophy.

### Clinical and echocardiographic characteristics of different LV geometrical patterns

In Table 4, Normal geometry and concentric remodelling LV patterns were present in patients with mean age around 58 years while concentric hypertrophy and eccentric hypertrophy patterns were present in patients with mean age above 60 years. With increasing age, abnormal LV geometrical patterns present itself in the order of concentric remodelling then concentric hypertrophy to eccentric hypertrophy (p<0.001).

**Table 4:** Clinical and echocardiographic characteristics of different LV geometrical patterns

**Clinical and echocardiographic variables (±SD)**	**Left ventricular geometrical patterns**				**p value**
	**Normal**	**Concentric remodelling**	**Concentric hypertrophy**	**Eccentric hypertrophy**	
Age (years)	57.62 ± 9.93	57.53 ± 9.99	61.19 ± 11.79	68.44 ± 10.25	<0.001
Male [n (%)]	36 (42.9)	12 (14.3)	16 (19.0)	20 (23.8)	0.003
Female [n(%)]	32 (22.4)	33 (23.1)	48 (33.4)	30 (21.0)	
Age at diagnosis (years)	54.85 ± 10.09	54.02 ± 8.84	55.17 ± 12.33	55.88 ± 11.13	0.865
BMI (kg/m^2^)	25.97 ± 4.250	27.20 ± 4.99	29.72 ±6.01	27.60 ±5.206	0.001
BSA (m^2^)	1.74 ± 0.17	1.74 ± 0.15	1.78 ± 0.19	1.74 ± 0.15	0.581
DBP (mmHg)	94.71 ± 10.14	98.67 ± 1.40	100.62 ± 10.97	95.20 ± 7.62	0.003
SBP (mmHg)	150.59 ± 14.24	166.67 ± 16.65	169.69 ± 20.39	159.60 ± 18.40	<0.001
MAP (mmHg)	113.33 ± 8.43	121.33 ± 11.53	123.65 ± 12.83	116.67 ± 10.034	<0.001
HTN duration (years)	2.76 ± 4.33	3.51 ± 3.32	6.02 ± 4.37	12.56 ± 8.41	<0.001
IVSWTd (cm)	0.96 ± 0.13	1.05 ± 0.11	1.59 ± 0.21	1.18 ± 0.14	<0.001
LVPWTd (cm)	0.87 ± 0.08	1.02 ± 0.10	1.47 ± 0.16	0.98 ± 0.098	<0.001
LVIDd (cm)	4.76 ± 0.29	4.35 ± 0.21	4.90 ± 0.34	5.41 ± 0.30	<0.001
LVIDs (cm)	3.23 ± 0.39	2.98 ± 0.27	3.36 ± 0.41	3.96 ± 0.38	<0.001
FS	32.53 ± 5.30	31.53 ± 4.92	31.72 ± 5.71	26.96 ± 4.27	<0.001
RWT (cm)	0.37 ± 0.04	0.47 ± 0.04	0.60 ± 0.08	0.36 ± 0.031	<0.001
LV mass (g)	150.00 ± 25.39	153.20 ± 19.71	326.12 ± 74.14	232.32 ± 25.38	<0.001
LVMI-BSA (g/m^2^)	86.59 ± 14.80	88.56 ± 11.22	187.22 ± 51.72	134.48 ± 25.84	<0.001
LVMI-height^2.7^ (g/m^2.7^)	40.32 ± 7.97	42.47 ± 5.41	94.47 ± 25.66	65.52 ± 13.37	<0.001

HTN=hypertension, BMI=Body Mass Index, BSA=Body surface area, DBP=Diastolic blood pressure, SBP=Systolic blood pressure, MAP=Mean arterial pressure, IVSWTd=Interventricular septal wall thickness at end-diastole, LVPWTd=LV posterior left ventricular wall at end-diastole, LVIDd=LV internal diameter at end-diastole, LVIDs=LV internal diameter at end-systole, FS=fractional shortening, RWT=Relative wall thickness , LVMI-BSA=Left Ventricular mass indexed to Body Surface Area, LVMI-height 2.7=left ventricular mass indexed to height 2.7.

Normal geometry is more frequent in male than female (42.9% vs. 22.4%) while concentric remodelling and concentric hypertrophy is more frequent in female than male (23.1% vs. 14.3% and 33.6% vs. 19.0% respectively). Eccentric hypertrophy did not differ much between male and female (23.8% vs. 21.0%). The difference in presentation of LV geometrical patterns with sex is statistically significant (p=0.003).

Abnormal LV geometrical pattern presentation with body mass index was present in patients who are at least overweight (BMI >25kg/m2). Concentric hypertrophy was present in patients who have higher mean BMI compared to patients with concentric remodeling and eccentric hypertrophy (29.72 vs. 27.20 and 27.60kg/m^2^ respectively).

Normal geometrical pattern was present in patients who have relatively smaller BMI (25.97kg/m^2^) compared to other geometrical patterns. The difference in body mass index and LV geometrical pattern presentation was significant (p=0.001).

There was a progressive increase in mean systolic, diastolic and mean arterial blood pressure in patients with normal geometry to patients with concentric hypertrophy, followed by decline in patients with eccentric hypertrophy (p<0.05).

LV geometrical patterns presentation change with duration of hypertension significantly (p<0.001), being lower in normal geometry and highest in eccentric hypertrophy (mean duration increased from 2.76 for normal to 12.56 years for eccentric hypertrophy).

There was a significant difference regarding the interventricular septal wall thickness at end-diastole, left ventricular posterior wall thickness at end diastole, relative wall thickness, left ventricular mass indexed to body surface are and height^2.7^ dimension presentation in different LV geometrical patterns, increasing from normal through concentric hypertrophy and then declining in eccentric hypertrophy (p<0.001).

The left ventricular internal diameters during diastole and systole were relatively higher in patients with normal geometrical than with concentric remodelling patterns but increased from patients with concentric remodeling to eccentric hypertrophy patterns. This difference was statistically significant (p<0.001).

The left ventricular fractional shortening was normal for normal geometrical, concentric remodeling and concentric hypertrophy geometrical patterns and was mildly lower for patients with eccentric hypertrophy.

### Clinical functional correlates of different LV geometrical patterns

As shown in Table 5, regarding clinical functional correlates of different LV geometrical patterns, chest tightness, exertional dyspnoea, paroxysymal nocturnal dyspnoea, lower limb oedema, palpitations and general body fatigue were more common in patients with eccentric hypertrophy and in patients with concentric hypertrophy. These features were almost absent in patients with concentric remodelling and normal LV geometry. This difference was statistically significant (p<0.001).

**Table 5:** Clinical functional correlates of different LV geometrical patterns

**Clinical features**	**Total [(n%)]**	**LV geometrical pattern**				**p value**
		**NG**	**CR**	**CH**	**EH**	
		**[n (%)]**	**[n (%)]**	**[n (%)]**	**[n (%)]**	
Dyspnoea at rest	5 (2.2)	0 (0)	0 (0)	0 (0)	5 (100.0)	<0.001
Chest tightness	13 (5.7)	2 (15.4)	0 (0)	1 (7.7)	10 (76.9)	<0.001
Exertional dyspnoea	31 (13.7)	0 (0)	1 (3.2)	6 (19.4)	24 (77.4)	<0.001
Paroxysmal nocturnal dyspnoea	13 (5.7)	0 (0)	0 (0)	3 (23.1)	10 (76.9)	<0.001
Lower limb oedema	22 (9.7)	0 (0)	0 (0)	6 (27.3)	16 (72.7)	<0.001
Palpitation	15 (6.6)	0 (0)	0 (0)	1 (6.7)	14 (93.3)	<0.001
General body fatigue	32 (14.1)	4 (12.5)	0 (0)	8 (25)	20 (62.5)	<0.001

NG=normal geometry, CR=concentric remodelling, CH=concentric hypertrophy, EH=eccentric hypertrophy.

### Determinants of left ventricular hypertrophy in hypertensive patients

As is shown in Table 6, the independent determinants of LVH after a step wise logistic regression analysis were age of the patient, systolic blood pressure, duration of hypertension and body mass index.

**Table 6:** Determinants of left ventricular hypertrophy in hypertensive patients

**Variables**									
		**B**	**S.E.**	**Wald**	**Df**	**p-value**	**Exp(B)**	**95.0% C.I.for EXP(B)**	
								**Lower**	**Upper**
Step 1^a^	HTN duration	0.209	0.035	35.435	1	0	1.233	1.151	1.321
	Constant	−1.138	0.22	26.752	1	0	0.321		
Step 2^b^	SBP	0.032	0.009	13.841	1	0	1.032	1.015	1.05
	HTN duration	0.22	0.036	37.238	1	0	1.246	1.161	1.338
	Constant	−6.363	1.441	19.492	1	0	0.002		
Step 3^c^	SBP	0.033	0.009	13.151	1	0	1.033	1.015	1.052
	HTN duration	0.227	0.037	37.031	1	0	1.255	1.166	1.35
	BMI	0.112	0.032	12.476	1	0	1.118	1.051	1.19
	Constant	−9.669	1.843	27.514	1	0	0		
Step 4^d^	Age	0.049	0.017	8.06	1	**0.005**	1.05	1.015	1.086
	SBP	0.032	0.009	11.48	1	**0.001**	1.032	1.014	1.052
	HTN duration	0.202	0.037	29.632	1	**0**	1.224	1.138	1.316
	BMI	0.147	0.036	17.129	1	**0**	1.159	1.081	1.242
	Constant	−13.343	2.453	29.582	1	0	0		

HTN duration=duration of hypertension, SBP=systolic blood pressure, DBP=diastolic blood pressure, BMI=body mass index.

a. Variable(s) entered on step 1: duration of Hypertension.

b. Variable(s) entered on step 2: Systolic blood pressure.

c. Variable(s) entered on step 3: Body mass Index.

d. Variable(s) entered on step 4: age.

## Discussion

In this study abnormal LV geometry was detected in 70% of patients while Aje [11] found 72% of newly diagnosed hypertensive patients to have abnormal LV geometry. Akintunde [12] found abnormal LV geometry in 72% of treated hypertensive patients. In this study, the distribution of different abnormal LV geometrical patterns was 19.8%, 28.2%, 22% for concentric remodeling, concentric hypertrophy and eccentric hypertrophy respectively. Aje [11] found the distribution of abnormal LV geometry to be 26%, 28% and 18% for concentric remodeling, concentric hypertrophy and eccentric hypertrophy respectively, while Akintunde [12] found the distribution to be 60.1%, 5.3% and 6.9% for concentric remodeling, eccentric hypertrophy and concentric hypertrophy respectively. The variation can partly be explained by difference in echocardiographic cut-off values for defining LVH and intrinsic population characteristics of a given geographical location.

Duration of hypertension markedly influenced the type of LV geometry with normal LV geometry predominating in early hypertension and abnormal geometrical patterns predominating in late hypertension, as is found also by Akintunde [12]. During hypertension the various cardiac morphological adaptations occur depending on the predominating stimuli. The type of LV geometry relates to the duration of hypertension and age of the patient mostly depending on the predominating type of stimuli i.e. pressure overload in early relatively ‘younger’ hypertensive patients while volume overload predominate in late and relatively ‘elderly’ hypertensive patients.

With regard to LVH, age of the patient, systolic blood pressure, duration of hypertension and body mass index were strong independent determinants of LVH. This was also shown in a Russian study [6].

Regarding blood pressure and duration of hypertension, there was significant change characterized by progressive increase in mean systolic, mean diastolic and mean arterial blood pressure from patients with normal geometry to patients with concentric hypertrophy, followed by decline in patients with eccentric hypertrophy overtime [12,13]. The studied echocardiographic parameters i.e. the interventricular septal wall thickness at end-diastole, left ventricular posterior wall thickness at end diastole, relative wall thickness, left ventricular mass indexed to body surface and height^2.7^ increased significantly from normal through concentric hypertrophy and then declining in eccentric hypertrophy.

This is because in patients with concentric remodeling or concentric hypertrophy usually the pressure overload predominates rather than volume overload stimuli. With pressure overload the wall tension is high trying to counteract the high peripheral resistance and usually the cardiac myocyte hypertrophy typical of concentric hypertrophy. With time, the hypertrophied cardiac muscle becomes maladapative and fatigues for reasons not known and so to overcome the volume overload the chambers dilate and cardiac output is decreased and resultant reduced systolic and diastolic pressure in eccentric hypertrophy.

## Conclusions

About 70% of hypertensive patients at KCMC which is a tertiary hospital in northern Tanzania had abnormal LV geometry, whereby 19.8% had concentric remodelling, 28.2% concentric hypertrophy and 22% eccentric hypertrophy.

The left ventricular systolic function by fractional shortening was worse in patients with eccentric hypertrophy who were also more likely to have clinical features of heart failure than patients with other types of geometrical patterns.

Hypertensive patients are not a homogenous population and characterization into different left ventricular phenotypes is important to stage an appropriate management and monitor prognosis.

## Competing interests

The authors declare that they have no any competing interests.

## Authors' contributions

**LSK:** first author of this work, involved in the early conception of the idea, proposal development, data analysis, manuscript writing and submitting to the publishing journal. **VPM:** highly contributed to the study concept, design, global and direct supervision of data collection and report writing. **HD:** was the focal person responsible for doing echocardiograms. **HM:** was involved in teaching basics hands-on technique on echocardiography to the main author GK: was involved in statistical analysis. **MD:** involved in proposal writing. **BH:** was involved in supervisory role in study concept, manuscript writing and critical review of the work at all stages. All authors read and approved the final manuscript.

## Pre-publication history

The pre-publication history for this paper can be accessed here:

http://www.biomedcentral.com/1471-2261/12/109/prepub

## References

[B1] Rosamond W Flegal K Furie K Go A Greenlund K Haase N Heart disease and stroke statistics–2008 update: a report from the American Heart Association Statistics Committee and Stroke Statistics Subcommittee Circulation 2008 117 4 e25 e146 18086926 10.1161/CIRCULATIONAHA.107.187998

[B2] Callow AD Cardiovascular disease 2005–the global picture Vascul Pharmacol 2006 45 5 302 307 10.1016/j.vph.2006.08.010 17074537

[B3] Ciolac EG Bocchi EA Bortolotto LA Carvalho VO Greve JM Guimaraes GV Haemodynamic, metabolic and neuro-humoral abnormalities in young normotensive women at high familial risk for hypertension J Hum Hypertens 2010 24 12 814 822 10.1038/jhh.2010.21 20237500

[B4] Ganau A Devereux RB Roman MJ de SG Pickering TG Saba PS Patterns of left ventricular hypertrophy and geometric remodeling in essential hypertension J Am Coll Cardiol 1992 19 7 1550 1558 10.1016/0735-1097(92)90617-V 1534335

[B5] Devereux RB Alonso DR Lutas EM Gottlieb GJ Campo E Sachs I Echocardiographic assessment of left ventricular hypertrophy: comparison to necropsy findings Am J Cardiol 1986 57 6 450 458 10.1016/0002-9149(86)90771-X 2936235

[B6] Conrady AO Rudomanov OG Zaharov DV Krutikov AN Vahrameeva NV Yakovleva OI Prevalence and determinants of left ventricular hypertrophy and remodelling patterns in hypertensive patients: the St. Petersburg study Blood Press 2004 13 2 101 109 10.1080/08037050410031855 15182113

[B7] de Ramirez SS Enquobahrie DA Nyadzi G Mjungu D Magombo F Ramirez M Prevalence and correlates of hypertension: a cross-sectional study among rural populations in sub-Saharan Africa J Hum Hypertens 2010 24 12 786 795 10.1038/jhh.2010.14 20220771

[B8] Cuspidi C Esposito A Negri F Sala C Masaidi M Giudici V Studies on left ventricular hypertrophy regression in arterial hypertension: a clear message for the clinician? Am J Hypertens 2008 21 4 458 463 10.1038/ajh.2007.85 18369363

[B9] Antoniucci D Seccareccia F Menotti A Dovellini EV Prati PL Rovelli F Prevalence and correlates of echocardiographic determined left ventricular hypertrophy in 2318 asymptomatic middle-aged men: the ECCIS project. Epidemiolgia e Clinica della Cardiopatia Ischemica Silente G Ital Cardiol 1997 27 4 363 369 9199953

[B10] Coca A Gabriel R De-la FM Lopez-Sendon JL Fernandez R Sagastagoitia JD The impact of different echocardiographic diagnostic criteria on the prevalence of left ventricular hypertrophy in essential hypertension: the VITAE study. Ventriculo Izquierdo Tension Arterial Espana J Hypertens 1999 17 10 1471 1480 10.1097/00004872-199917100-00016 10526909

[B11] Aje A Adebiyi AA Oladapo OO Dada A Ogah OS Ojji DB Left ventricular geometric patterns in newly presenting Nigerian hypertensives: an echocardiographic study BMC Cardiovasc Disord 2006 6 4 10.1186/1471-2261-6-4 16426452 PMC1361785

[B12] Akintunde A Akinwusi O Opadijo G Left ventricular hypertrophy, geometric patterns and clinical correlates among treated hypertensive Nigerians Pan Afr Med J 2010 4 8 21119993 10.4314/pamj.v4i1.53602 PMC2984315

[B13] Davila DF Donis JH Odreman R Gonzalez M Landaeta A Patterns of left ventricular hypertrophy in essential hypertension: should echocardiography guide the pharmacological treatment? Int J Cardiol 2008 124 2 134 138 10.1016/j.ijcard.2007.01.089 17467083

